# Contribution of Chromosome 14 to Exercise Capacity and Training Responses in Mice

**DOI:** 10.3389/fphys.2019.01165

**Published:** 2019-09-13

**Authors:** Michael P. Massett, Sean M. Courtney, Seung Kyum Kim, Joshua J. Avila

**Affiliations:** Department of Health and Kinesiology, Texas A&M University, College Station, TX, United States

**Keywords:** chromosome substitution strain, genetic, inbred mice, treadmill running, quantitative trait loci (QTL), sex differences

## Abstract

Quantitative trait loci for exercise capacity and training-induced changes in exercise capacity were identified previously on mouse Chromosome 14. The aim of this study was to further investigate the role of Chromosome 14 in exercise capacity and responses to training in mice. Exercise phenotypes were measured in chromosome substitution strain mice carrying Chromosome 14 from the PWD/PhJ donor strain on the genetic background of a host C57BL/6J (B6) strain (B6.PWD14). Eight week old female and male mice from both strains completed a graded exercise test to exhaustion to assess intrinsic or baseline exercise capacity. A separate group of 12-week old female and male mice, randomly assigned to sedentary control (SED) or exercise training (EX) groups, completed a graded exercise test before and after a 4-week exercise training period. EX mice completed a 4-week training program consisting of treadmill running 5 days/week, 60 min/day at a final intensity of approximately 65% of maximum. For intrinsic exercise capacity, exercise time and work were significantly greater in female and male B6.PWD14 than sex-matched B6 mice. In the training study, female B6.PWD14 mice had higher pre-training exercise capacity than B6 mice. In contrast, there were no significant differences for pre-training exercise capacity between male B6 and B6.PWD14 mice. There were no significant strain differences for responses to training. These data demonstrate that PWD/PhJ alleles on Chromosome 14 significantly affect intrinsic exercise capacity. Furthermore, these results support continued efforts to identify candidate genes on Chromosome 14 underlying variation in exercise capacity.

## Introduction

Low exercise capacity is a risk factor and predictor of future disease comparable to elevated systolic blood pressure, obesity, diabetes, and smoking (Myers et al., 2002; Kokkinos et al., 2008). Improving cardiorespiratory fitness through increased exercise training can significantly reduce the risk of all-cause mortality (Blair et al., 1995; Kokkinos et al., 2010), regardless of the level of initial fitness (Erikssen et al., 1998). However, responses to exercise training are highly variable such that some individuals can show minimal or no improvements in exercise capacity, i.e., cardiorespiratory fitness (Lortie et al., 1984; Kohrt et al., 1991; Bouchard et al., 1999, 2011). Genetics contribute significantly to individual variation in baseline or intrinsic exercise capacity and the response to training in humans, with heritability estimates of ∼50% for each phenotype (Bouchard et al., 1998, 1999). Although recent genome-wide studies have identified novel candidate genes linked to exercise responses (Timmons et al., 2010; Bouchard et al., 2011), these results have yet to be replicated due to the difficulty in conducting exercise intervention studies in humans (Hagberg et al., 2011).

As an alternative, genetically defined model organisms such as inbred or selectively bred strains of rodents also are being utilized to investigate the genetic basis for exercise capacity and responses to training. Based on differences in treadmill running performance among inbred strains of rodents, heritability estimates for exercise capacity range from 31 to 73% (Barbato et al., 1998; Koch et al., 1998; Lightfoot et al., 2001; Lerman et al., 2002). Estimated heritability for responses to training in inbred rodents is significant, but more variable (13–58%) (Koch and Britton, 2001; Massett and Berk, 2005; Avila et al., 2017). Similarly, heritability for maximal running distance is approximately 45–60% in rats selectively bred for intrinsic exercise capacity and their F_2_ generation offspring (Ren et al., 2013). Collectively, these data suggest that variation in exercise capacity and responses to training in mice and rats are significantly influenced by genetics.

To identify novel genes influencing exercise phenotypes, several genome wide mapping strategies have been used to define genomic regions linked to intrinsic exercise capacity and responses to training in rats and mice (Ways et al., 2002; Lightfoot et al., 2007; Massett et al., 2009, 2015; Courtney and Massett, 2012). For example, in a genome wide linkage scan using an F_2_ population derived from inbred FVB/NJ (FVB) and C57BL/6J (B6) mice, we identified significant quantitative trait loci (QTL) for intrinsic exercise capacity and a suggestive QTL for the change in exercise capacity (i.e., training response) on Chromosome 14 (Massett et al., 2009). Although these QTL were novel for rodents, comparative genomic mapping indicates several linkage markers for VO_2__max_ in the sedentary state or changes in VO_2__max_ or mean power output in humans fall within the 1.5 LOD intervals for these QTL (Bouchard et al., 2000, 2011; Rico-Sanz et al., 2004; Timmons et al., 2010). In addition, a single nucleotide polymorphism (SNP) in the gene mitochondrial intermediate peptidase (MIPEP) is associated with training responses in humans also map to regions sytenic with QTL on Chromosome 14 (Timmons et al., 2010; Rice et al., 2012). A SNP in that gene was used to predict training responses in humans based on baseline gene expression in skeletal muscle (Timmons et al., 2010). This collection of evidence suggests that mouse Chromosome 14 should be considered for more detailed analyses of the genetic basis for endurance exercise capacity and responses to training.

In the current study we used a chromosome substitution strain (CSS) to assess the contribution of Chromosome 14 to endurance exercise capacity and responses to training. CSS mice are made by substituting a single chromosome from a donor inbred strain on the genetic background of a host inbred strain (recipient) (Nadeau et al., 2000; Singer et al., 2004). Phenotypic differences between the recipient or background strain and CSS mice support the presence of a QTL on the substituted chromosome for the phenotype being measured. Kvedaras et al. (2017) used a CSS strain derived from C57BL/6J and A/J strains to demonstrate that genes on Chromosome 10 from the A/J strain contribute to low exercise capacity in inbred A/J mice. CSS mice used in the current study carry Chromosome 14 from the PWD/PhJ strain on the C57BL/6J background (Gregorová et al., 2008). PWD/PhJ mice are genetically diverse relative to the inbred C57BL/6J strain. There are approximately 127,000 SNPs on mouse Chromosome 14 where C57BL/6J and PWD/PhJ strains differ (Mouse Phenome Database^1^) (Frazer et al., 2007). Approximately 400 of these SNPs result in a nucleotide substitution that alters the amino acid sequence of the protein (coding non-synonymous), suggesting that the function of one or more proteins might differ between strains. In addition, we previously reported the C57BL/6J strain have low exercise capacity compared to the PWD/PhJ strain (Courtney and Massett, 2012) and changes in exercise capacity in response to endurance training are greater in PWD/PhJ mice than C57BL/6J (Avila et al., 2017). Heart mass, muscle mass, and skeletal muscle fiber type differences between PWD/PhJ and C57BL/6J mice have also been reported (Kilikevicius et al., 2013; Avila et al., 2017). Thus, the PWD/PhJ and C57BL/6J strains have both genetic and phenotypic differences that support the use of CSS derived from these strains as a model for investigating the genetic basis for variation in exercise capacity.

Utilizing this CSS model, the aim of this study was to test the hypothesis that exercise capacity and the change in exercise capacity in response to training would be higher in CSS mice than the background strain. Data supporting this hypothesis would confirm the importance of Chromosome 14 in genetic regulation of intrinsic exercise capacity and training responses in mice. It would also support future studies localize small genomic regions and identify candidate genes for exercise capacity and responses to training.

## Materials and Methods

### Animals

This study was carried out in accordance with the recommendations of the National Institutes of Health guidelines for the care and use of laboratory animals. The protocol was approved by the Institutional Animal Care and Use Committee at Texas A&M University. Inbred C57BL/6J (B6), and Chromosome 14 substitution mice, C57BL/6J-Chr 14 ^PWD/Ph/ForeJ^ (B6.PWD14) were purchased from Jackson Laboratory (Bar Harbor, ME, United States). Mice were group housed depending on sex and strain in standard cages, allowed food (Standardized Laboratory Rodent Diet) and water *ad libitum*, and maintained on a 12 h light:dark schedule at 22–24°C. Mice were allowed 1 week to acclimate after arrival.

### Exercise Capacity

Intrinsic or baseline exercise capacity was measured in 8-week-old, young adult male and female B6 and B6.PWD14 mice. Immediately following two consecutive days of familiarization, mice completed two graded exercise tests on a motorized rodent treadmill (Columbus Instruments, Columbus, OH, United States) separated by 48 h (Courtney and Massett, 2012; Avila et al., 2017). After a 9 min warm-up at 9.0 m/min and 0° grade, the grade was increased 5° every 9 min to a maximum of 15°. Speed increased 2.5 m/min from a starting speed of 10 m/min every 3 min until exhaustion. Exercise capacity was estimated using time (minutes) and work (kg⋅m).

### Exercise Training

Twelve-week old male and female B6 and B6.PWD14 mice were randomly assigned to exercise training (EX) or sedentary control (SED) groups (*n* = 5-6/group) prior to completing exercise performance tests (as above). Exercise tests were performed before and after the training period. EX mice ran at a workload of 65% of maximum, 5 days/wk for 60 min/day for 4 weeks (Massett et al., 2009; Avila et al., 2017). SED mice performed pre- and post-exercise tests and were handled weekly but not made to run. SED mice repeated the familiarization protocol before the post-training exercise tests. Twelve-week old adult mice were used for the training study to avoid any contributions of growth and/or maturation to the training responses that might occur in young adult (e.g., 8 week old) mice (Murach et al., 2017).

### Tissue Samples

At least 24 h after the last exercise performance test, mice were anesthetized by intraperitoneal injection of ketamine (80 mg/kg) – xylazine (5 mg/kg) cocktail and euthanized by exsanguination. Heart and soleus, plantaris, and gastrocnemius muscles were excised and wet weights (in mg) obtained prior to freezing to calculate tissue to body mass ratios as markers of skeletal muscle and cardiac hypertrophy. Body mass was measured before each exercise performance test and prior to terminal surgery.

### Statistical Analysis

Data are presented as mean ± SD. Two-way ANOVA was used to determine strain by sex comparisons for exercise and anthropometric variables in the inbred strain and CSS mice followed by a Tukey *post hoc* test. Differences between SED and EX groups within each strain were compared by Student’s unpaired *t*-tests. Multiple regression analysis was used to determine the relationship between variables (e.g., sex, strain, body mass, heart mass) and intrinsic exercise capacity or the change in exercise capacity. Statistical significance was set at *P* < 0.05. Analyses were performed using JMP Pro 13.1.0 (SAS Institute, Inc., Cary, NC, United States) or Prism 5.0 (GraphPad Software, Inc., La Jolla, CA, United States). Effect sizes (Cohen’s *d*) for exercise phenotypes were calculated using mean differences between strains (Cohen, 1988). Thresholds were set as small, |d| ≤ 0.5; medium, |d| < 1.0; large, |d| < 1.5; and very large, |d| ≥ 1.5 (Labots et al., 2016).

## Results

### Intrinsic (Baseline) Exercise Capacity

**FIGURE 1:**
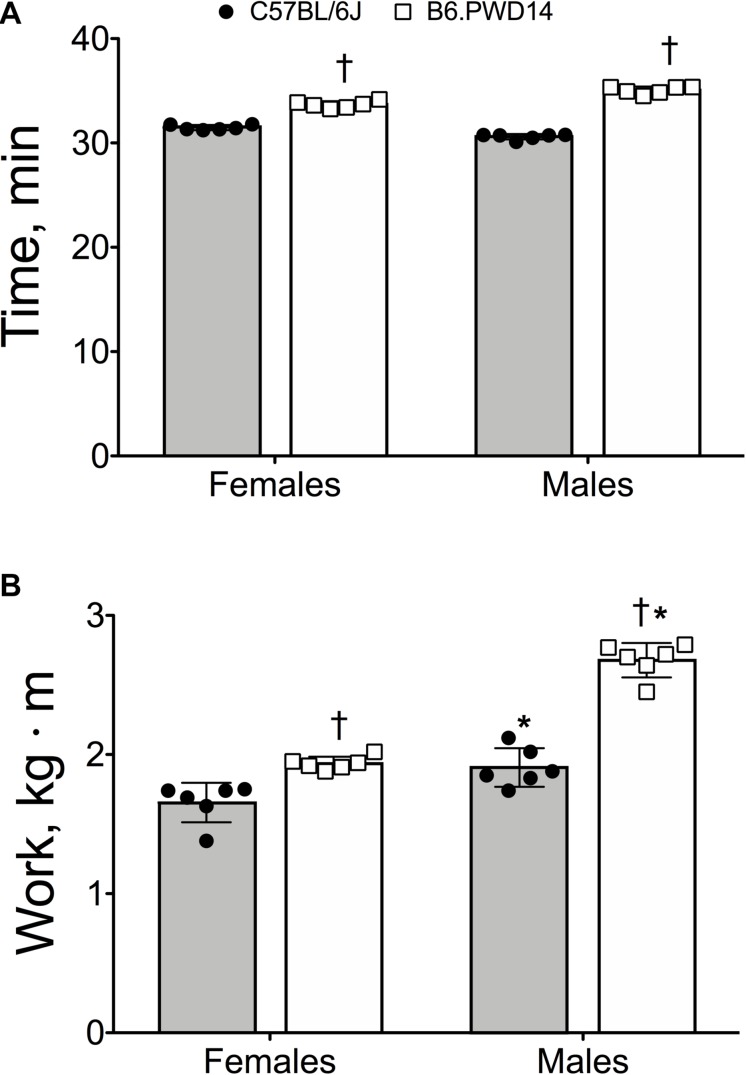
Endurance exercise capacity expressed as time **(A)** and work **(B)** in female and male inbred C57BL/6J and B6.PWD14 mice. The chromosome substitution strain (CSS) mice used in this study are based on the C57BL/6J background and carry Chromosome 14 from the PWD/PhJ (B6.PWD14) strain. All mice performed a graded run-to-exhaustion endurance exercise test; exercise capacity was expressed as time (minutes) and work (kg⋅m). Values are expressed as mean ± SD. *n* = 6 per group. ^∗^*P* < 0.05 significantly different from females of the same strain; ^†^*P* < 0.05 significant strain effect vs. B6.

Exercise time in B6.PWD14 mice was 11% higher than B6 mice (34.7 ± 0.8 vs. 31.4 ± 0.8 min, *P* < 0.0001). Time was similar between male and female mice (*P* = 0.056), but the strain by sex interaction was significant (*P* < 0.0001). For both sexes, exercise time was significantly higher in B6.PWD14 than B6 (Figure 1A). Effect sizes for time were *d* = 11.7 [95% CI = 6.4–15.3] in males and *d* = 3.9 [1.9–9.9] for females. B6.PWD14 mice performed ∼30% more work than B6 mice (2.31 ± 0.40 vs. 1.84 ± 0.09 kg⋅m, *P* < 0.0001). Within each strain, work was significantly greater in males than females (*P* < 0.0001). The strain-by-sex interaction was significant (*P* < 0.0001). Nevertheless, B6.PWD14 mice performed significantly greater work than B6 mice regardless of sex (Figure 1B). Effect sizes were *d* = 8.4 [4.4–11.0] for males and *d* = 3.5 [1.5–5.0] for females.

Significant main effects for strain were detected for plantaris muscle mass and plantaris mass-to-body mass ratio (Table 1). For both phenotypes, B6.PWD14 mice had significantly greater values than B6 mice. A main effect for sex was detected for most phenotypes with several significant strain-by-sex interactions (Table 1). Multiple regression analysis was used to identify factors associated with intrinsic exercise capacity. In addition to strain (β = 1.74, *SE* = 0.12, *P* < 0.0001, η^2^ = 0.91), gastrocnemius mass-to-body mass ratio (β = −0.57, *SE* = 0.19, *P* = 0.0087, η^2^ = 0.04) had a small, negative effect on intrinsic exercise capacity.

**TABLE 1:** Anthropometric phenotypes in female and male C57BL/6J and B6.PWD14 mice.

	**C57BL/6J**		**B6.PWD14**	
	**Female**	**Male**	**Female**	**Male**
Body mass, g	18.5 ± 1.4	22.8 ± 1.8^∗^	18.0 ± 0.4	22.7 ± 0.8^∗^
Heart mass, g	104 ± 5	122 ± 14^∗^	102 ± 4	115 ± 4^∗^
Soleus mass, g	8 ± 1	10 ± 1^∗^	8 ± 1	9 ± 1^∗^
Plantaris mass, g **^†^**	18 ± 1	20 ± 1^∗^	19 ± 1	22 ± 2^∗^
Gastrocnemius mass, g	113 ± 8	152 ± 15^∗^	127 ± 8	135 ± 6
HM:BM, mg/g	5.68 ± 0.51	5.03 ± 0.31	5.69 ± 0.15	5.03 ± 0.08
SM:BM, mg/g	0.41 ± 0.07	0.39 ± 0.03	0.42 ± 0.03	0.39 ± 0.02
PM:BM, mg/g **^†^**	0.97 ± 0.09	0.84 ± 0.05^∗^	1.03 ± 0.05	0.96 ± 0.08
GM:BM, mg/g	6.18 ± 0.72	6.26 ± 0.36	7.07 ± 0.46	5.90 ± 0.26^∗^

*B6.PWD14, CSS based on the C57BL/6J background and substituted Chromosome 14 from the PWD/PhJ strain; HM:BM, heart mass-to-body mass ratio; SM:BM, soleus mass-to-body mass ratio; PM:BM, plantaris mass-to-body mass ratio; GM:BM, gastrocnemius mass-to-body mass ratio. ^∗^*P* < 0.05 compared with females of the same strain; ^†^*P* < 0.05 B6.PWD14 significantly different from C57BL/6J; *n* = 6/group. Data were analyzed by two-way ANOVA (sex and strain) followed by Tukey *post hoc* test. All data are mean ± SD.*

### Exercise Training

In both strains, changes in exercise capacity were higher in EX than SED mice (B6.PWD14 EX = 3.7 ± 0.8 min, SED = 0.2 ± 1.0 min, *P* < 0.0001; B6 EX = 4.0 ± 1.2 min, SED = 0.8 ± 1.0 min, *P* < 0.0001) with no baseline differences. Within strains, there were no differences between groups for baseline work, but changes in work were higher in EX than SED mice (B6.PWD14 EX = 0.47 ± 0.15 kg⋅m, SED = 0.06 ± 0.12 kg⋅m, *P* < 0.0001; B6 EX = 0.50 ± 0.15 kg⋅m, SED = 0.10 ± 0.11 kg⋅m, *P* < 0.0001). Thus, 4 weeks of exercise training improved exercise capacity in EX mice from both strains. In B6.PWD14 mice, the change in body mass was significantly smaller in EX compared with SED mice (Supplementary Table 1) and heart mass-to-body mass ratio was significantly greater in B6.PWD14 EX than SED mice (Supplementary Table 1).

**FIGURE 2:**
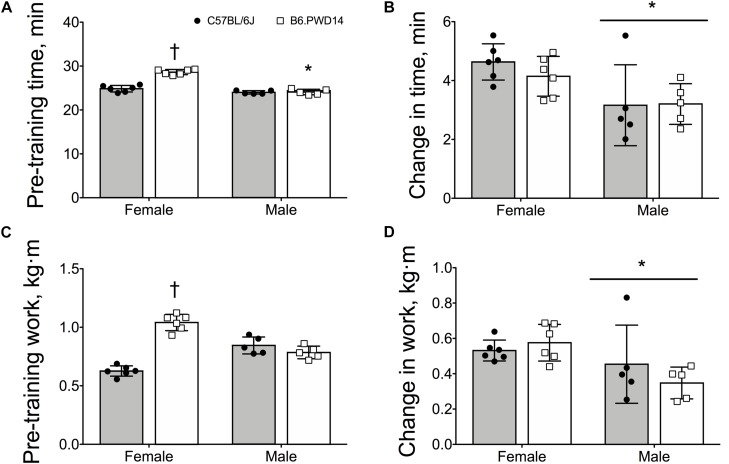
Pre-training exercise capacity and responses to training in female and male inbred C57BL/6J and B6.PWD14 mice. Pre-training exercise capacity and responses to training are expressed as time (minutes) **(A,B)** and work (kg⋅m) **(C,D)**. The 4-week exercise training program consisted of treadmill running 5 days/week, 60 min/day at a final intensity equivalent to approximately 65% of the maximal work-load (speed and incline) attained during the initial graded treadmill test. All mice performed a graded exercise test to exhaustion before and after 4 weeks of exercise training. B6.PWD14: CSS based on the C57BL/6J background and carry Chromosome 14 from the PWD/PhJ (B6.PWD14) strain. Values are expressed as mean ± SD. *n* = 5–6 per group. ^∗^*P* < 0.05 significantly different from females; ^†^*P* < 0.05 significant strain effect vs. B6.

In EX mice, there were significant main effects for strain (*P* < 0.0001) and significant strain-by-sex interactions (*P* < 0.0001) for intrinsic exercise time and work. Baseline time and work were significantly greater in female B6.PWD14 than female B6 (*P* < 0.0001) (Figures 2A,C). Cohen’s *d* = 5.7 [2.9–7.6] and *d* = 6.4 [3.3–8.6] for time and work, respectively. Conversely, baseline time and work were comparable between male B6.PWD14 and B6 mice (Figures 2A,C). Effect sizes were *d* = 0.2 [−1.0 to 1.4] for time and *d* = −0.9 [−2.1 to 0.5] for work.

There were no differences between strains for changes in time (*P* = 0.55, Figure 2B) or work (*P* = 0.58, Figure 2D). Changes in time (*P* < 0.0001) and work (*P* = 0.013) were greater in females than males. Effect sizes for change in time were *d* = −0.7 [−1.9 to 0.5] and *d* = 0.1 [−1.2 to 1.3] for females and males, respectively. For the change in work, *d* = 0.6 [−0.6 to 1.7] for females and *d* = −0.6 [−1.8 to 0.7] for males.

Body mass was similar between strains before training (*P* = 0.2, Table 2); however, there was a significant main effect for sex (*P* < 0.0001). Within each strain, body mass was greater in males than females. The change in body mass was significantly greater in B6 (1.0 ± 0.7 g) than B6.PWD14 (0.1 ± 0.5 g) (*P* = 0.0017). Changes in body mass were similar between male and female mice. There were no strain differences for other anthropometric phenotypes (Table 2).

**TABLE 2:** Anthropometric phenotypes in female and male C57BL/6J and B6.PWD14 mice from EX groups.

	**C57BL/6J**		**B6.PWD14**	
	**Female**	**Male**	**Female**	**Male**
Body mass, g				
Pre-training	19.2 ± 1.2	28.6 ± 1.3^∗^	19.8 ± 0.8	26.5 ± 2.3^∗^
Post-training	20.3 ± 0.8	29.5 ± 1.7^∗^	20.1 ± 1.1	26.2 ± 2.6^∗^,^†^
Change	1.1 ± 0.7	0.9 ± 0.7	0.4 ± 0.4	−0.3 ± 0.4^†^
Heart mass, g	106 ± 11	137 ± 7^∗^	102 ± 13	134 ± 20^∗^
Soleus mass, g	10 ± 1	14 ± 3^∗^	10 ± 2	12 ± 2
Plantaris mass, g	17 ± 2	26 ± 3^∗^	20 ± 3	24 ± 8
Gastrocnemius mass, g	118 ± 6	180 ± 32^∗^	104 ± 26	164 ± 18^∗^
HM:BM, mg/g	5.21 ± 0.51	4.67 ± 0.20	5.04 ± 0.51	5.13 ± 0.62
SM:BM, mg/g	0.47 ± 0.06	0.47 ± 0.11	0.50 ± 0.12	0.46 ± 0.06
PM:BM, mg/g	0.81 ± 0.10	0.88 ± 0.12	1.00 ± 0.18	0.94 ± 0.31
GM:BM, mg/g	5.80 ± 0.37	6.10 ± 0.96	5.14 ± 1.21	6.25 ± 0.53

*EX, all mice completed 4 weeks of exercise training; B6.PWD14, CSS based on the C57BL/6J background and substituted Chromosome 14 from the PWD/PhJ strain; HM:BM, heart mass-to-body mass ratio; SM:BM, soleus mass-to-body mass ratio; PM:BM, plantaris mass-to-body mass ratio; GM:BM, gastrocnemius mass-to-body mass ratio; ^∗^*P* < 0.05 compared with females of the same strain; ^†^*P* < 0.05 compared with C57BL/6J of the same sex, *n* = 5–6/group. Data were analyzed by two-way ANOVA (sex and strain) followed by Tukey *post hoc* test. All data are mean ± SD.*

Multiple regression analysis was used to determine the relation between intrinsic or baseline exercise capacity and anthropometric variables using all B6 and B6.PWD14 mice. Sex, strain, body mass, plantaris muscle mass, and plantaris mass-to-body mass ratio were all significantly related to intrinsic or baseline exercise capacity (Supplementary Table 2). Sex (18%), strain (13%), and body mass (14%) accounted for greatest variance in exercise capacity in this population. In EX group mice, sex, baseline exercise time, and soleus muscle mass were related to change in exercise time (Supplementary Table 2) with sex accounting for the greatest variance (54%) in the change in exercise capacity.

## Discussion

In the current study, we used two groups of CSS and inbred mice to investigate the influence of Chromosome 14 on exercise capacity and responses to training. For intrinsic exercise capacity, male and female mice carrying Chromosome 14 from PWD/PhJ strain on a B6 background have a significantly greater exercise capacity (time and work) than inbred B6 mice. Effect sizes for strain differences in exercise capacity were very large for both sexes. In separate groups of mice utilized to assess training responses, a significant difference in intrinsic exercise capacity between strains was observed in female mice. In contrast, there were no strain differences in changes in exercise capacity with training. These results suggest that one or more QTL on Chromosome 14 influences variation in intrinsic exercise capacity, but additional experiments are required to identify loci contributing to responses to exercise training.

The advantage of using CSS is that genetic variation is limited to one chromosome, thereby reducing complexity of the genetic interactions that might influence a trait in a mixed genetic background (Nadeau et al., 2000). In this study, B6.PWD14 mice ran 11% longer and performed 30% more work than B6 mice. The strain effect was very large (*d* ≥ 1.5) for both phenotypes. The greater exercise capacity in the B6.PWD14 mice suggests that the substituted chromosome shifted the phenotype toward the donor PWD/PhJ strain. Differences between B6.PWD14 and B6 strains for exercise time and work provide additional evidence that genetic factors on mouse Chromosome 14 are important for determining exercise capacity. Previously, we reported significant QTL for exercise time and work on Chromosome 14 using intercross populations derived from inbred mouse strains (Massett et al., 2009, 2015). Using CSS mice derived from A/J and C57BL/6J strains, suggestive QTL for time and work also were identified on Chromosome 14 (Courtney and Massett, 2014). Furthermore, several markers identified by linkage analysis for VO_2__max_ in the sedentary state and mean power output in humans (Bouchard et al., 2000; Rico-Sanz et al., 2004) map to QTL intervals on mouse Chromosome 14 for intrinsic exercise capacity in mice. Collectively, this evidence supports the presence of one or more QTL for intrinsic exercise capacity on this chromosome. Although utilizing CSS for genetic analyses can confirm and localize QTL to the chromosome level, there are > 900 protein-coding genes on mouse Chromosome 14 (Mouse Genome Informatics^2^). Therefore, linkage analysis using intercross or backcross offspring from B6 and B6.PWD14 strains or development of congenic strains is needed to reduce the QTL interval on this chromosome and facilitate candidate gene discovery for these exercise phenotypes.

Interestingly, in the older adult mice, strain differences were only observed in female mice. This finding implies that the genetic architecture underlying exercise capacity might be different between males and females. We previously reported a significant effect of sex on exercise capacity (Courtney and Massett, 2014; Massett et al., 2015) and the responses to training in mice (Massett et al., 2009, 2015). Furthermore, QTL for exercise capacity were identified in two separate cohorts of female mice that were not present in their male counterparts (Courtney and Massett, 2014; Massett et al., 2015). Lightfoot et al. (2007) also identified a suggestive QTL for endurance exercise capacity in female, but not male mice. Sex-specific QTL also have been reported for voluntary wheel running (Lightfoot et al., 2010) and exercise-related traits such as muscle mass (Nehrenberg et al., 2010; Carroll et al., 2011). Thus, some evidence supports the concept that genetic architecture for complex traits such as exercise capacity differs in males and females. Therefore, sex-specific affects should be considered when investigating the genetic regulation of exercise capacity and responses to training, which are known to differ between males and females.

Changes in exercise capacity with training vary between inbred mouse strains and are determined, in part, by genetics (Avila et al., 2017). In this study, mice from both strains increased exercise capacity by ∼15% in response to 4 weeks of exercise training. The change in exercise capacity in B6 mice is consistent with previous reports (Massett et al., 2009; Gibb et al., 2016; Avila et al., 2017). In our previous study, training responses in PWD/PhJ mice were nearly double that of inbred B6 (Avila et al., 2017). Therefore, we expected B6.PWD14 mice to have a greater response to training than the host B6 strain, if PWD/PhJ alleles on Chromosome 14 contributed to this response. However, there were no differences between strains. These data suggest that alleles on the donor PWD/PhJ chromosome do not influence exercise training responses on the host B6 background, the QTL effect is too small to be detected in the current experiment, or the substituted chromosome contains alleles that elicit phenotypic effects in opposite directions (i.e., increase and decrease training responses) (Nadeau et al., 2012). In contrast, we identified a suggestive QTL for exercise training responses on Chromosome 14 in F_2_ mice derived from inbred FVB/NJ and C57BL6J strains (Massett et al., 2009). That QTL interval is concordant with regions of the human genome linked to changes in VO_2__max_ with training (Bouchard et al., 2000; Rico-Sanz et al., 2004). Single nucleotide polymorphisms associated with changes in oxygen consumption at maximal and submaximal workloads in humans also map to regions syntenic with the QTL on Chromosome 14 (Timmons et al., 2010). Thus, data from mice and humans support the presence of a QTL on Chromosome 14 for exercise training responses. However, the complexity of the phenotype might necessitate additional animals or alternative animal models to more thoroughly investigate the genetic basis for exercise training responses.

Heart and skeletal muscle from the lower hindlimb were collected to identify phenotypes such as muscle mass that might be associated with intrinsic exercise capacity or responses to training and linked to the action of potential candidate genes. Soleus, plantaris, and gastrocnemius muscles are utilized during treadmill running in rodents (Armstrong and Laughlin, 1983, 1984) and have been shown to undergo adaptations with exercise training (Kemi et al., 2002; Massett and Berk, 2005; Bye et al., 2008). In the current study, plantaris muscle mass and plantaris mass-to-body mass ratio were significantly associated with intrinsic exercise capacity (Supplementary Table 2). Combined they accounted for approximately 14% of the variation in intrinsic exercise capacity. In addition, plantaris muscle mass and plantaris muscle mass-to-body mass ratio were significantly different between B6 and B6.PWD14 strains (Table 1). These differences suggest QTL for plantaris muscle mass and plantaris muscle mass-to-body mass ratio also reside on Chromosome 14. Several of the genes on Chromosome 14 are associated with cardiac or skeletal muscle structure/function, including myosin, heavy polypeptide 6, cardiac muscle, alpha (*Myh6*), myosin, heavy polypeptide 7, cardiac muscle, beta (*Myh7)*, myozenin 1 (*Myoz1)*, and sarcoglycan, gamma (dystrophin-associated glycoprotein) (*Sgcg)*. Three of these genes (*Myh6, Myh7, Myoz1*) contain SNPs that differ between B6 and PWD/PhJ strains. None of the SNPs are known to change the amino acid sequence of the protein suggesting that further investigation of candidate genes should not be limited to these three. However, the strain differences in exercise capacity and plantaris muscle mass support further consideration of genes on Chromosome 14 that influence hindlimb muscle mass as potential candidate genes for both muscle mass and intrinsic exercise capacity.

In summary, the results of this study provide additional evidence that one or more genetic loci on Chromosome 14 contribute to variation in exercise capacity. Concordance among human and mouse studies suggest that the genes underlying variation in exercise capacity might be conserved between species. However, the exact molecular targets have yet to be determined. In contrast, the support for genetic loci on Chromosome 14 contributing to exercise training responses is not as strong and requires further investigation.

## Data Availability

All datasets generated for this study are included in the manuscript and/or the Supplementary Files.

## Ethics Statement

This study was carried out in accordance with the recommendations of the National Institutes of Health guidelines for the care and use of laboratory animals. The protocol was approved by the Institutional Animal Care and Use Committee at Texas A&M University.

## Author Contributions

JA, SC, SK, and MM conceived and designed the experiments, drafted, edited, and revised manuscript, and approved the final version of the manuscript. JA, SK, and SC performed the experiments. JA, SC, and MM analyzed the data.

## Conflict of Interest Statement

The authors declare that the research was conducted in the absence of any commercial or financial relationships that could be construed as a potential conflict of interest.

## Footnotes

1 https://phenome.jax.org/snp/retrievals

2 http://www.informatics.jax.org
