# Supplementary material for: Contribution of Chromosome 14 to Exercise Capacity and Training Responses in Mice
Source: Front Physiol. 2019 Sep 13;10:1165. doi: 10.3389/fphys.2019.01165 (PMC6753330; doi:10.3389/fphys.2019.01165)
Supplement: Supplementary file 1 [file Table_1.DOCX]

| **Supplemental Table 1.** Anthropometric phenotypes in SED and EX groups from C57BL/6J and B6.PWD14 strains. | | | | |
| --- | --- | --- | --- | --- |
|  | C57BL/6J | | B6.PWD14 | |
|  | SED | EX | SED | EX |
| Body mass, g |  |  |  |  |
| Pre-training | 24.8 ± 5.7 | 23.5 ± 5.0 | 22.6 ± 2.5 | 22.8 ± 3.8 |
| Post-training | 25.7 ± 4.8 | 24.5 ± 5.0 | 24.2 ± 3.2 | 22.9 ± 3.7 |
| Change | 1.0 ± 1.4 | 1.0 ± 0.7 | 1.6 ± 1.2 | 0.1 ± 0.5 * |
| Heart mass, g | 126.3 ± 26.8 | 120.1 ± 18.7 | 111.5 ± 10.9 | 116.5 ± 23.3 |
| Soleus mass, g | 11.3 ± 3.2 | 11.5 ± 3.2 | 10.7 ± 2.7 | 11.0 ± 2.5 |
| Plantaris mass, g | 21.4 ± 5.1 | 20.7 ± 5.6 | 23.2 ± 3.3 | 22.0 ± 5.8 |
| Gastrocnemius mass, g | 143.9 ± 28.7 | 145.9 ± 38.5 | 123.3 ± 19.3 | 130.8 ± 38.2 |
| HM:BM, mg/g | 4.92 ± 0.60 | 4.96 ± 0.47 | 4.64 ± 0.38 | 5.08 ± 0.53 * |
| SM:BM, mg/g | 0.44 ± 0.08 | 0.47 ± 0.08 | 0.44 ± 0.06 | 0.48 ± 0.09 |
| PM:BM, mg/g | 0.83 ± 0.13 | 0.84 ± 0.68 | 0.96 ± 0.08 | 0.97 ± 0.23 |
| GM:BM, mg/g | 5.62 ± 0.72 | 5.94 ± 0.68 | 5.10 ± 0.51 | 5.64 ± 1.09 |

SED, sedentary control mice; EX, all mice completed 4 weeks of exercise training; B6.PWD14, CSS based on the C57BL/6J background and substituted Chromosome 14 from the PWD/PhJ strain; HM:BM, heart mass-to-body mass ratio; SM:BM, soleus mass-to-body mass ratio; PM:BM, plantaris mass-to-body mass ratio; GM:BM, gastrocnemius mass-to-body mass ratio;
*, P < 0.05 compared with SED of the same strain by unpaired t-test, n = 11-12/group; data from male and female mice were combined for each group. All data are mean ± SD.
