# Supplementary material for: Contribution of Chromosome 14 to Exercise Capacity and Training Responses in Mice
Source: Front Physiol. 2019 Sep 13;10:1165. doi: 10.3389/fphys.2019.01165 (PMC6753330; doi:10.3389/fphys.2019.01165)
Supplement: Supplementary file 2 [file Table_2.DOCX]

| **Supplemental Table 2.** Regression analysis of exercise time and response to training in adult male and female C57BL/6J and B6.PWD14 mice. | | | | | | |
| --- | --- | --- | --- | --- | --- | --- |
| Phenotype | R^2^ | p-value | Variable | Beta coefficient (± error) | p-value | Eta^2^ |
| Baseline exercise time | 0.78 | < 0.0001 | Sex | 2.13 ± 0.37 | < 0.0001 | 0.18 |
|  |  |  | Strain | 0.86 ± 0.17 | < 0.0001 | 0.13 |
|  |  |  | Body mass, g | 0.57 ± 0.11 | < 0.0001 | 0.14 |
|  |  |  | Plantaris mass, mg | -0.43 ± 0.13 | 0.0014 | 0.06 |
|  |  |  | PM:BM, mg/g | 11.38 ± 3.02 | 0.0005 | 0.08 |
|  |  |  |  |  |  |  |
| Change in time | 0.57 | 0.0015 | Sex | 1.14 ± 0.24 | 0.0002 | 0.54 |
|  |  |  | Baseline exercise time, min | -0.24 ± 0.10 | 0.03 | 0.13 |
|  |  |  | Soleus mass, mg | 0.13 ± 0.07 | 0.08 | 0.08 |
| PM:BM, plantaris mass-to-body mass ratio. | | | | | | |
